# Bioprotection Potential of *Lacticaseibacillus rhamnosus* LRH01 and *Lactiplantibacillus plantarum* LP01 against Spoilage-Associated *Penicillium* Strains in Yoghurt

**DOI:** 10.3390/molecules28217397

**Published:** 2023-11-02

**Authors:** Ce Shi, Susanne Knøchel

**Affiliations:** 1 School of Food and Biological Engineering, Jiangsu University, Xuefu Road 301, Zhenjiang 212013, China; 2 Section of Food Microbiology and Fermentation, Department of Food Science, Faculty of Science, University of Copenhagen, Rolighedsvej 26, 1958 Frederiksberg C, Denmark; skn@food.ku.dk

**Keywords:** *Lacticaseibacillus rhamnosus*, *Lactiplantibacillus plantarum*, yoghurt biopreservation, *Penicillium*, antifungal mechanisms, manganese depletion

## Abstract

*Penicillium* spp. are considered a major spoilage fungus in dairy products. Due to the growing concerns over food safety issues and the demand for “clean label” food products from consumers, the use of lactic acid bacteria (LAB) as a bioprotective tool to control fungal spoilage of dairy products appears to be a promising alternative. Here, the antifungal activities of ten LAB cultures against five dairy-spoilage-associated *Penicillium* strains were studied in a model system, and the most potent bioprotective cultures were further tested in yoghurt. *Lacticaseibacillus rhamnosus* (*L. rhamnosus*) LRH01 and *Lactiplantibacillus plantarum* (*L. plantarum*) LP01 exhibited potent antifungal efficacy at low concentrations. The inhibitory effects of cell-containing fermentates (C-fermentates), cell-free fermentates (CF-fermentates), and volatiles produced by the two cultures were tested in a yoghurt serum medium. The C-fermentates showed antifungal effects, while the removal of cells from C-fermentates led to decreased antifungal activities. Volatiles alone displayed some antifungal efficiency, but less than the fermentates. In a yoghurt matrix, the specific effect of manganese depletion by the bioprotective cultures on mold growth was investigated. Here, the LAB cultures could completely suppress the growth of molds, while addition of manganese partially or fully restored the mold growth, demonstrating that manganese depletion played a key role in the antifungal activity of the tested LAB cultures in the yoghurt matrix. Both *L. plantarum* LP01 and *L. rhamnosus* LRH01 showed efficient antifungal activities in the yoghurt serum, while *L. rhamnosus* LRH01 exhibited the most potent inhibitory effects on *Penicillium* strains when added during the processing of the yoghurt with subsequent storage at 7 °C for 22 days. Our findings suggested that *L. rhamnosus* LRH01 could be a promising bioprotective culture for yoghurt biopreservation.

## 1. Introduction

Fermented dairy products such as yoghurt are of high economic importance. Although yoghurt is generally considered to be microbiologically stable, it may be subjected to contaminations with acid-resistant spoilage fungi at the processing site as well as by the consumers. *Penicillium* is the most frequently and abundantly isolated genus among the dairy-associated spoilage molds, including species such as *Penicillium roqueforti* (*P. roqueforti*), *Penicillium glabrum* (*P. glabrum*), and *Penicillium commune* (*P. commune*) [1,2,3,4]. Mold spoilage represents a significant factor that may impact the stability and commercial value of dairy products, and spoilage not only leads to substantial food waste and economic losses worldwide but may also result in adverse effects on human health due to mycotoxin production in some cases [2]. To avoid mold problems in the dairy industry, an extensive range of hurdle technologies have been used, including chemical preservatives such as sodium benzoate, potassium sorbate, and potassium benzoate [5]. With increasing fungal resistance to these chemical preservatives and the growing demand for natural, less processed, healthy, or preservative-free “clean label” food products, the use of bioprotective cultures as a promising alternative to control unwanted organisms, and thus extend food shelf life, has attracted much attention in recent years [6,7].

Many lactic acid bacteria (LAB) species are regarded as obvious candidates for bioprotection due to a long history of safe use as indicated by their GRAS (Generally Recognized as Safe) status [8] and inclusion in the “Qualified Presumption of Safety” (QPS) list in Europe [9,10]. In addition, several LAB cultures, including *Lactiplantibacillus plantarum* (*L. plantarum*), *Lacticaseibacillus rhamnosus* (*L. rhamnosus*), and *Lacticaseibacillus casei* strains, have been observed to display antifungal activity, thereby potentially extending the shelf life of dairy products [6,9,11]. It was, for example, recently found that the growth of *P. commune* was inhibited by *L. plantarum* 885, 897, and 299v, indicating that these *L. plantarum* cultures could be promising candidates for the biopreservation of Cheddar cheese [12]; another study discovered that *L. rhamnosus* exhibited a strong inhibitory effect on fungal growth on semi-hard cheese after 6 weeks of ripening at 10 °C [13].

Concerning the antifungal mode of action, considerable efforts have been devoted to elucidating the mechanisms behind the antifungal potential of LAB. Numerous metabolites identified from LAB cultures have been seen to exhibit antifungal activities, such as organic acids including lactic acid and acetic acid, fatty acids, proteinaceous compounds including bacteriocins and bacteriocin-like substances, and other compounds with low or medium molecular weight mass including volatiles, such as diacetyl [14]. It has, however, been noticed that the concentrations of these antifungal metabolites produced by LAB cultures in situ have typically been significantly lower than their corresponding minimal inhibitory concentrations (MICs) [15]. Other antifungal mechanisms have therefore been investigated, and more recently, competitive exclusion through manganese depletion was uncovered as the main antifungal mechanism behind the antifungal activity of the *L. paracasei* and an *L. rhamnosus* strains in yoghurt [16]. The addition of manganese with increasing concentrations (up to 6 mg/L) positively correlated with the mold and yeast growth. Our previous study also demonstrated that 0.001–0.1 mM of manganese supplementation could partially or fully restore the growth of *Penicillium* and *Mucor* strains, otherwise inhibited by *L. plantarum* LP37 [17].

The aim of the current study is to test the bioprotective potential of several cultures against a panel of five common dairy-spoilage-associated *Penicillium* strains, and investigate the contributing role of LAB fermentates and volatiles to the antifungal activity in a model system as well as the specific role of manganese depletion in a yoghurt matrix and finally in a challenge test in order to determine their potential as bioprotective cultures for yoghurt.

## 2. Results

### 2.1. Antifungal Potential Evaluation

**Figure 1:**
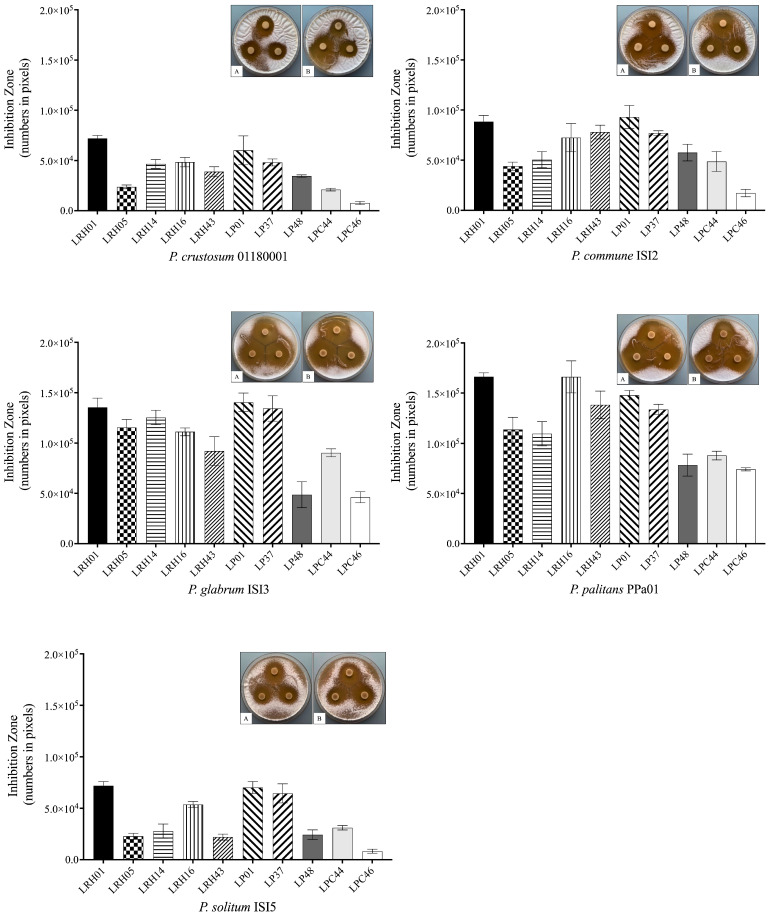
Antifungal potential of ten LAB cultures (10^7^ CFU/mL) on MRS agar overlaid with a suspension of five *Penicillium* strains. Results were expressed as the inhibition zone area (number in pixels). The values represent mean ± SD. Images showed the inhibition zones of *L. rhamnosus* LRH01 (**A**) and *L. plantarum* LP01 (**B**) against each mold after incubation for 3 days at 25 °C, respectively. LRH01, LRH05, LRH14, LRH16, and LRH43 were *L. rhamnosus* strains; LP01, LP37, and LP48 were *L. plantarum* strains; LPC44 and LPC46 were *L. paracasei* strains.

The antifungal potential of the ten LAB cultures against the five dairy-spoilage-associated *Penicillium* strains (Table 1) is shown in Figure 1. All the LAB cultures were active against the tested molds, producing a clear inhibition zone area, although large variations were observed after incubation at 25 °C for 3 days. In general, *L. rhamnosus* and *L. plantarum* seemed more effective at inhibiting mold growth compared with the *L. paracasei* cultures (*L. paracasei* LPC44 and *L. paracasei* LPC46). In particular, *L. rhamnosus* LRH01, *L. plantarum* LP01, and *L. plantarum* LP37 at 10^7^ CFU/mL exhibited stronger antifungal activity against the *Penicillium* strains, while *L. plantarum* LP48 and two *L. paracasei* strains were less active. With regards to the target organisms, the *Penicillium* strains displayed various degrees of sensitivity towards the LAB cultures with *P. glabrum* ISI3 and *P. palitans* PPa01 being more sensitive, and *P. crustosum* 01180001 and *P. solitum* ISI5 more resistant to the LAB cultures.

### 2.2. Minimal Inoculum Levels of LAB Cultures to Achieve Fungal Inhibition

After incubation at 25 °C for 3 days, the growth of all the tested molds was strongly inhibited by these LAB cultures at high inoculum levels such as 10^7^ CFU/mL (Table 2). However, large variations in the antifungal potential were observed, with the minimal inoculum levels ranging from 10^4^ to 10^7^ CFU/mL. Two out of the ten LAB cultures presented efficient growth-inhibitory effects, in some cases at concentrations down to 10^4^ CFU/mL, namely *L. rhamnosus* LRH01 and *L. plantarum* LP01. For example, the growth of *P. glabrum* ISI3, *P. solitum* ISI5, and *P. palitans* PPa01 was inhibited by *L. rhamnosus* LRH01 at 10^4^ CFU/mL, and *L. plantarum* LP01 at 10^4^ CFU/mL suppressed the growth of *P. solitum* ISI5, *P. palitans* PPa01, and *P. crustosum* 01180001. In contrast, the minimal inoculum levels of *L. plantarum* LP48 and *L. paracasei* LPC46 against all the tested molds were 10^7^ CFU/mL, demonstrating that higher concentrations of these cultures were required to completely inhibit fungal growth. After incubation for 5 days, the mold growth was still inhibited by the LAB cultures at 10^7^ CFU/mL. In general, when concentrations below 10^7^ CFU/mL were applied, the effect on mold growth decreased significantly or even disappeared, illustrating that although some LAB cultures could suppress mold growth at a low concentration, a higher concentration (e.g., 10^7^ CFU/mL) was required to achieve extended inhibitory action.

Based on the above results, *L. rhamnosus* LRH01 and *L. plantarum* LP01 were selected for the following experiments due to their efficient antifungal activities.

### 2.3. Antifungal Activity of Selected LAB Cultures in Yoghurt Serum

#### 2.3.1. Interaction of *Penicillium* Strains and C-Fermentates with Live Cells

**Figure 2:**
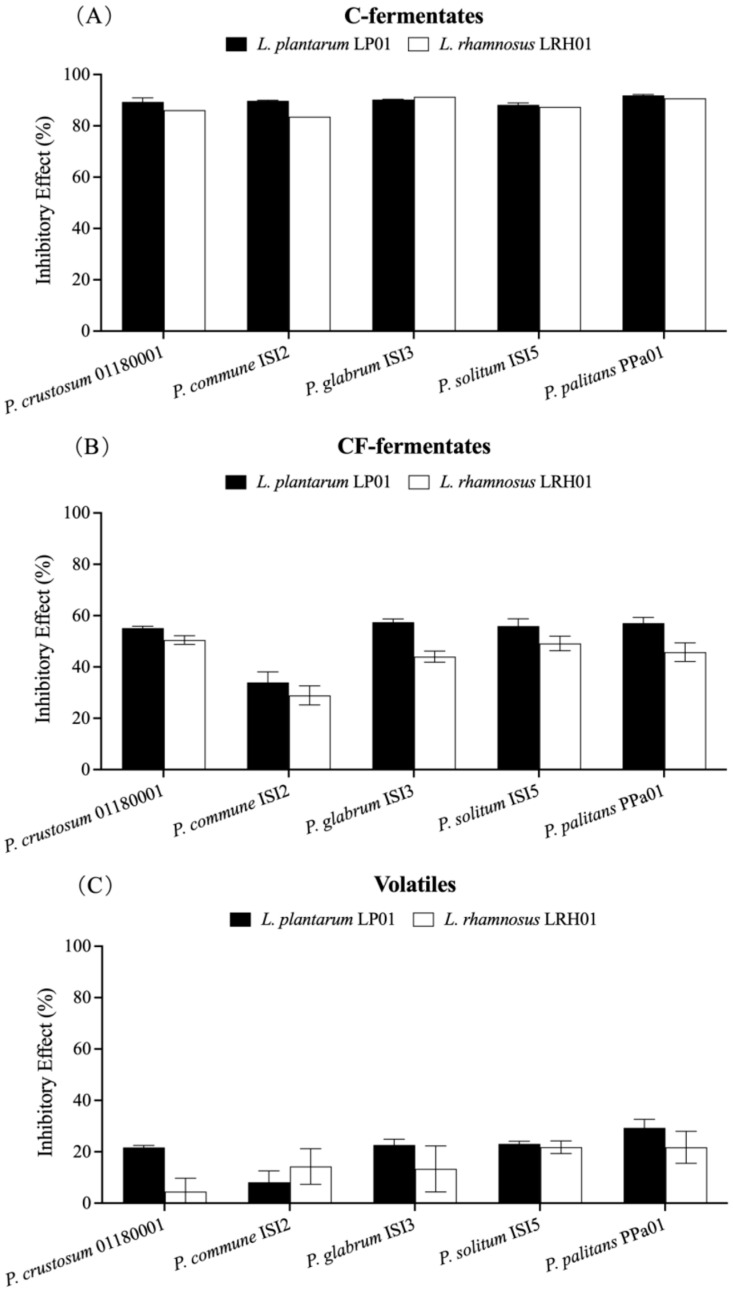
Inhibitory effects (%) of C-fermentates (**A**), CF-fermentates (**B**), and volatiles (**C**), respectively, produced by *L. plantarum* LP01 and *L. rhamnosus* LRH01 in yoghurt serum against five *Penicillium* strains. Control groups were prepared by spotting each mold suspension on the plates prepared by mixing un-inoculated MRS broth with agar. Error bars represent the standard error of the mean of three replicates.

To evaluate the contribution of LAB metabolites to the overall antifungal activity of these LAB cultures, the inhibitory effects of cell-containing fermentates (C-fermentates), cell-free fermentates (CF-fermentates), and the volatiles on the growth of five *Penicillium* strains were tested. Moreover, yoghurt serum was prepared as a yoghurt-mimicking model to replace the laboratory medium. Results in this test showed that the C-fermentates of *L. rhamnosus* LRH01 and *L. plantarum* LP01 in yoghurt serum exhibited potent antifungal activities against the five *Penicillium* strains, with the inhibitory effects ranging from 83% to 92% after incubation for 5 days at 25 °C, respectively (Figure 2A). Among the five *Penicillium* strains, the *P. glabrum* ISI3 and *P. palitans* PPa01 were more sensitive towards the C-fermentates of *L. rhamnosus* LRH01 and *L. plantarum* LP01 with inhibitory effects up to 90%. *P. commune* ISI2 was less sensitive to the C-fermentate of *L. rhamnosus* LRH01, the inhibitory effects were around 83%, followed by *P. crustosum* 01180001 and *P. solitum* ISI5. In the case of *L. plantarum* LP01, *P. solitum* ISI5 was less sensitive to the C-fermentate.

#### 2.3.2. CF-Fermentates

The antifungal activities of CF-fermentates of *L. rhamnosus* LRH01 and *L. plantarum* LP01 are shown in Figure 2B. Although the inhibitory effects significantly decreased after removal of cells from C-fermentates of the two cultures, large variations in mold-growth inhibition were still observed. In general, the inhibitory effects of the two CF-fermentates on the five *Penicillium* strains ranged from 26% to 59%. In comparison with *L. rhamnosus* LRH01, the CF-fermentate of *L. plantarum* LP01 exhibited more potent antifungal effects. The inhibition could be due to both the preformed metabolites produced by the LAB cultures in the yoghurt and/or a depletion of the limiting nutrients or trace elements by the LAB cultures [17].

#### 2.3.3. Contribution of Volatiles to the Antifungal Activity

**Figure 3:**
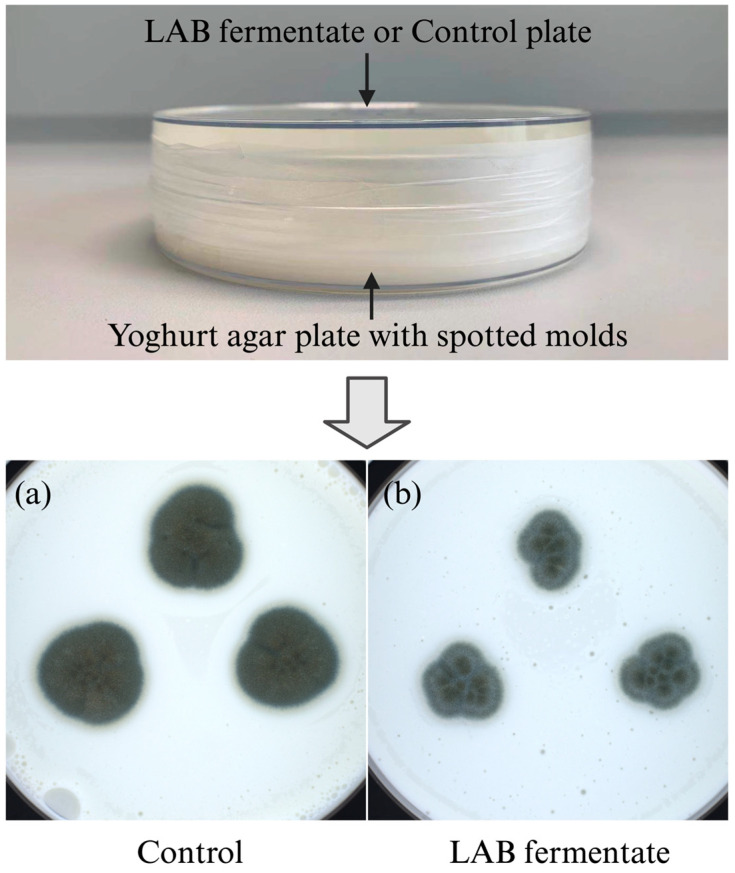
An example of “Plate-on-Plate” system. Influence of volatiles produced by *L. rhamnosus* LRH01 fermentate (top plate) on the growth of *P. commune* ISI2 spotted in triplicates on a yoghurt plate (bottom plate). Growth of mold on bottom yoghurt agar plate with either a non-inoculated control plate (yoghurt serum agar plate) on top (**a**) or a LAB inoculated agar plate on top (**b**).

To test the antifungal effects of the volatiles produced by LAB cultures, a plate-on-plate system was employed in this assay (Figure 3). Compared with the LAB fermentates with or without live cells, the inhibitory effects of volatiles produced by *L. rhamnosus* LRH01 or *L. plantarum* LP01 were much weaker (Figure 2C), ranging from 4% to 31%. Generally, volatiles from *L. plantarum* LP01 showed stronger inhibitory effects compared with those from *L. rhamnosus* LRH01. *P. commune* ISI2 only responded weakly to both LAB cultures, indicating low susceptibility to the volatiles, while *P. palitans* PPa01, on the other hand, was relatively sensitive. These data here were consistent with the results of the C-fermentate test. In summary, the results suggest that the volatiles produced by *L. rhamnosus* LRH01 and *L. plantarum* LP01 contribute, but only to a limited extent, to the overall antifungal activity.

### 2.4. Effect of Manganese on the Antifungal Activity

**Figure 4:**
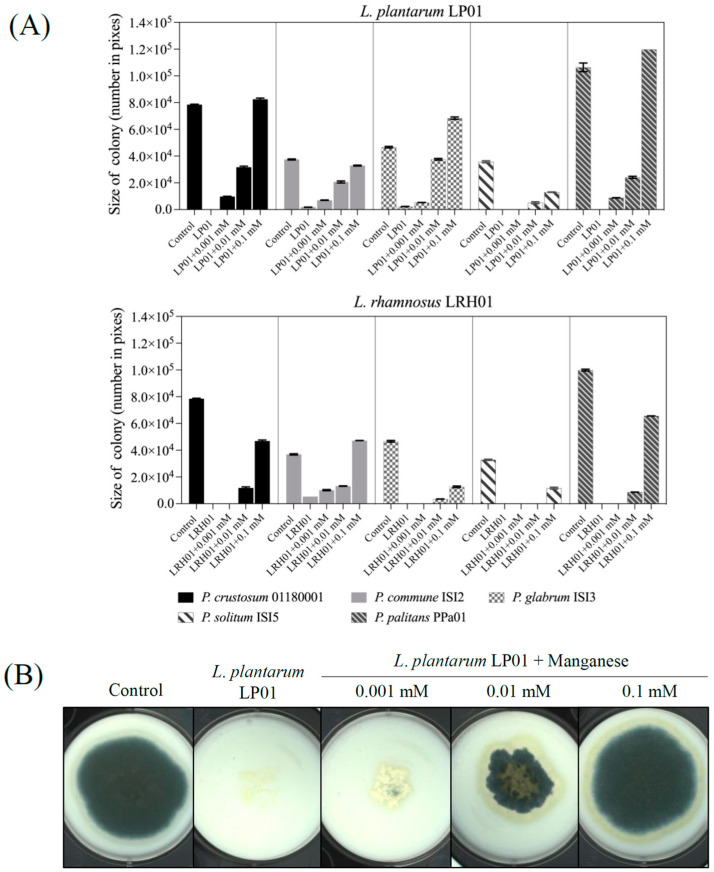
(**A**) Changes in mold colony size (number in pixels) on the yoghurt agar plates with different Mn levels. The values were expressed as the mean values of three replicates. (**B**) Images of mold growth on yoghurt plates showing *L. plantarum* LP01 and *P. crustosum* 01180001 as example. Manganese in different concentrations was added as indicated. The spoilage molds were added in concentrations of 200 spores/spot. The plates were incubated at 25 °C for 5 days. LP01 and LRH01 indicates *L. plantarum* LP01 and *L. rhamnosus* LRH01, respectively.

Here, a commercial plain yoghurt was used as a matrix to ensure a realistic manganese level. The *Penicillium* strains showed various levels of sensitivity when grown with *L. plantarum* LP01 or *L. rhamnosus* LRH01 on yoghurt-agar plates with or without manganese (Figure 4A). In comparison to the control groups, the growth of the five *Penicillium* strains was markedly inhibited by both *L. plantarum* LP01 and *L. rhamnosus* LRH01 in yoghurt. However, mold growth was partially or fully restored when manganese was added in increasing concentrations from 0.001 mM to 0.1 mM. For example, *P. crustosum* 01180001 growth was completely inhibited by *L. plantarum* LP01 in yoghurt, but the addition of manganese at 0.1 mM resulted in fully restored mold growth (Figure 4B). Although supplementation of manganese also restored the mold growth in yoghurt prepared with *L. rhamnosus* LRH01, it is worth noting that, in comparison to *L. plantarum* LP01, the *L. rhamnosus* LRH01 seemed more efficient at removing manganese since higher amounts of manganese were required to restore mold growth, especially in the case of *P. solitum* ISI5 and *P. palitans* PPa01 where growth could not be restored even after 0.01 mM manganese was added. In the case of *L. plantarum* LP01, the growth of most tested molds could be partially restored when 0.001 mM of manganese was added, while in the case of *L. rhamnosus* LRH01, 0.01 mM of manganese was required.

### 2.5. Challenge Test in Yoghurt

**Figure 5:**
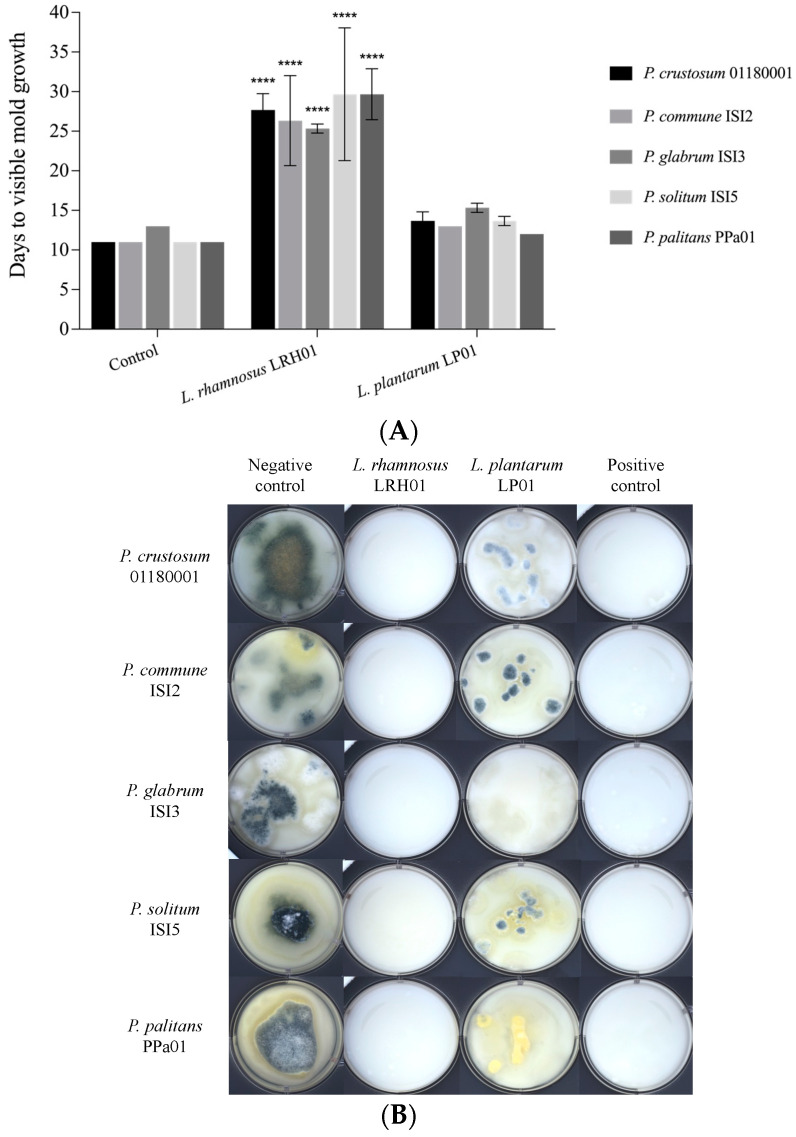
(**A**) Days of visible mold growth delay caused by *L. rhamnosus* LRH01, *L. plantarum* LP01, and LR4PD (positive control). Wells with indicator molds but without either *L. rhamnosus* LRH01 or *L. plantarum* LP01 served as negative controls. The values were expressed as the mean values of three replicates. Significant statistical differences compared with the control group were all at **** *p* < 0.0001. (**B**) Images of yoghurt with spotted molds after incubation for 22 days at 7 °C.

The challenge test was carried out by simulating the expected processing and storage conditions more closely, using yoghurt produced with the test strains added together with the starter cultures and simulating surface contamination of the finished yoghurt during storage at 7 °C. Here, *L. rhamnosus* LRH01 exhibited strong antifungal activity against the five tested *Penicillium* strains in yoghurt. The negative control groups without bioprotective cultures showed visible growth of these *Penicillium* strains after 9–13 days. The yoghurt samples with *L. rhamnosus* LRH01, however, delayed growth of the five *Penicillium* strains for a further 12–18 days, while *L. plantarum* LP01 delayed the mold growth for only 1–2 days (Figure 5A). As shown in Figure 5B, after incubation for 22 days at 7 °C, all the negative control groups were overgrown with the target molds, while there was almost no visible mold growth in the yoghurt samples inoculated with *L. rhamnosus* LRH01. In contrast, although mold growth was partly inhibited by *L. plantarum* LP01 in comparison with yoghurt without added test cultures, the *L. plantarum* LP01 in yoghurt did not exhibit a similar efficacy as seen previously in MRS medium.

## 3. Discussion

In the present study, LAB cultures with potent antifungal activities against five dairy-associated *Penicillium* strains were first screened out in laboratory media. Subsequently, the relative role of actively growing cells versus bioactive metabolites was investigated in a yoghurt serum matrix, and the role of manganese competitive exclusion in the antifungal effect was tested in plain yoghurt. Finally, selected LAB cultures were used as bioprotective cultures in a challenge test. At first, two methods were used to screen the most efficient mold inhibitory LAB cultures in MRS medium: an assessment of the antifungal potential at the same LAB inoculum level (10^7^ CFU/mL) and an evaluation of the minimal inoculum levels needed for inhibition. At 10^7^ CFU/mL, the most efficient strains belong to the species of *L. rhamnosus* and *L. plantarum*, and, in particular, to the strains of *L. rhamnosus* LRH01, *L. rhamnosus* LRH16, *L. plantarum* 01, and *L. plantarum* 37. Several *L. plantarum* as well as *L. rhamnosus* strains have also been found to have inhibitory effects on fungal growth in previous studies [9,12,13,18]. All ten LAB cultures exhibited inhibitory effects against dairy-associated spoilage molds, but the inhibitory efficacy is highly strain dependent. Among the tested strains here, *L. rhamnosus* LRH01 and *L. plantarum* 01 showed the most potent antifungal activity at low concentrations (10^4^ CFU/mL). Therefore, *L. rhamnosus* LRH01 and *L. plantarum* 01 were selected for the further investigations.

Several previous studies have looked at the antifungal metabolites produced by LAB in the supernatant [18,19,20,21,22]. In order to investigate the role of antifungal metabolites in the antifungal effects of LAB cultures during inhibition in a yoghurt scenario, the sensitivity of the five *Penicillium* strains towards LAB fermentates with or without live cells in yoghurt serum, and towards volatiles, was explored. As expected, the fermentates with live cells of *L. rhamnosus* LRH01 and *L. plantarum* 01 significantly inhibited *Penicillium* strains growth. However, the inhibitory effect decreased after the removal of the live cells from the fermentates, indicating that the presence of live cells in the fermentates increased the inhibitory effects. A recent review summarized many of the compounds associated with antifungal effects including organic acids, short- and long-chain fatty acids such as 3-phenyllactic acid, 4-hydroxyphenyllactic acid, and indole-lactic acid, as well as proteinaceous compounds such as bacteriocins. In most cases, the inhibition assays have been conducted in model systems [23]. In addition, the contribution of volatiles produced by LAB cultures to the antifungal activity has been reported in recent years. Volatile compounds, including acetic acid responsible of the antifungal activity, were identified from *L. plantarum* cultures [24] and it has previously been highlighted that the main volatile compound produced by *L. paracasei* DGCC 2132 is diacetyl, which exhibited antifungal activity against *P. solitum* DCS 302 and *Penicillium* sp. nov. DCS 1541 [14]. In addition, the specific effect of diacetyl against *P. commune* and *P. crustosum* in yoghurt has been reported. The MIC was 32 μg/mL, and the antifungal mechanism was related to the integrity of the cell membrane and oxidative stress [25]. Recently, the application of volatile compounds produced by LAB cultures has been mentioned as a promising strategy for the prevention and control of fungal contamination in food products. A volatile compound, 1-pentanal, produced by a *Lactobacillus curvatus* strain showed excellent inhibition against *A. flavus* in red pepper powder, indicating that this volatile could be a promising biopreservative agent to prevent fungal contamination [26]. In the present study, however, volatiles produced by *L. plantarum* LP01 and *L. rhamnosus* LRH01 in yoghurt serum were only moderately effective in suppressing the growth of *Penicillium* strains, indicating that volatiles alone were a contributing but not a main factor in this system.

Competitive exclusion through manganese depletion was recently found to be the main mechanism in several LAB cultures for restraining the growth of spoilage fungi [16]. Manganese is an essential component in a variety of enzymes with physiological functions and acts as a cofactor in some metalloenzymes [27,28,29]. In the current study, it was found that manganese played a key role in the antifungal activity of the tested LAB cultures in a yoghurt matrix since the addition of manganese could restore mold growth in a concentration-dependent manner. More manganese was required in the case of *L. rhamnosus* LRH01 than was seen for *L. plantarum* LP01. For *L. plantarum* LP01, the addition of 0.001 mM of manganese in the treatment group could restore the mold growth of e.g., *P. solitum* ISI5, while 0.1 mM of manganese was required to restore mold growth in the case of *L. rhamnosus* LRH01. Therefore, *L. rhamnosus* LRH01 supposedly possesses a stronger manganese depletion capacity in comparison with *L. plantarum* 01, resulting in a more potent antifungal activity in yoghurt. LAB have been shown to be able to take up the manganese in an environment or culture medium through two major uptake systems which are composed of the Natural Resistance-Associated Macrophage Protein (NRAMP)-type transporter MntH (under acidic conditions) and the ABC-type transporter SitABC (mainly active at neutral pH) [30]. Recently, manganese transporter genes (*mnth1, mnt2,* and *mnt3*) and the manganese transport operon *mntr*, were identified in *L. plantarum* CCFM436, and MntH 1–3, were speculated to be the potential manganese importers which were negatively regulated by MntR [31]. Similar research also reported that the expression of the manganese transporter, MntH1, encoded by the *mnth1* gene, was significantly higher in *L. rhamnosus* and *L. paracasei*, facilitating the exhaustive manganese removal in fermented milk [16]. Further studies should be performed to evaluate the manganese transporter system of potential bioprotective cultures in products with different matrix properties, oxygen availability, and pH and at different temperatures.

To validate the application of *L. rhamnosus* LRH01 and *L. plantarum* LP01 as bioprotective cultures in dairy products, a challenge test was conducted in yoghurt where the strains had been added during production. Here, *L. rhamnosus* LRH01 caused a significant visible mold growth delay at 7 °C, while there was very limited mold growth delay observed in the case of *L. plantarum* LP01. Furthermore, *L. rhamnosus* LRH01 still displayed antifungal activity in yoghurt after incubation for 22 days, which was in line with the results in the positive control group. These results demonstrated that *L. rhamnosus* LRH01 could be a promising bioprotective culture in yoghurt production. It is worth noting that *L. plantarum* 01 exhibited stronger inhibitory effects on the *Penicillium* strains when the test with the yoghurt serum took place at an elevated room temperature, while in yoghurt, where the strains were part of both the fermentation and the later chill storage, the *L. rhamnosus* LRH01 showed a more potent inhibition. It is possible that these differences partly reflect how well these cultures are adapted to the temperatures encountered both during fermentation and later during storage since growth rates and metabolic activity may differ [2]. It therefore adds to the complexity of predicting mold sensitivity and efficacy of bioprotective cultures that impact factors such as matrix composition including manganese content and storage temperature should be taken into account.

## 4. Materials and Methods

### 4.1. Organisms and Chemicals

The frozen stock cultures of the 10 LAB cultures used in this study (Table 1) were stored at −80 °C in De Man, Rogosa, and Sharpe (MRS) broth with the addition of 20% glycerol. The LR4PD, composed of *L. rhamnosus* strains (commercial culture Lyofast supplied by Sacco S.r.l, Cadorago, Italy), was used as the positive control in the yoghurt challenge test. Five *Penicillium* strains used as indicator molds were isolated from freshly fermented dairy products (Table 1). Malt extract agar plates (MEA, pH 5.6 ± 0.2) prepared using 30 g/L malt extract, 5 g/L peptone, and 15 g/L agar were used as culture medium for mold growth at 25 °C for 7 days, and then the spore suspension of each mold was collected by centrifugation in malt extract broth (MEB, 17 g/L malt extract, 3 g/L peptone, pH 5.6 ± 0.2), and counted with a Malassez counting chamber. The suspension concentration of each mold was adjusted to 1.0 × 10^6^ spores/mL and subsequently stored at −80 °C for further use. Malt extract, peptone, and agar were obtained from Thermo Fisher Scientific Inc. (New York, NY, USA). Manganese chloride (MnCl_2_), purchased from Sigma-Aldrich (Schnelldorf, Germany), was used in the manganese depletion test. The commercial plain yoghurt (0.5% fat, Arla Foods, Viby, Demark) was obtained from the local supermarket (Copenhagen, Denmark).

### 4.2. Antifungal Activity of LAB Cultures

The antifungal effects of the ten LAB cultures against the *Penicillium* strains were determined based on the overlay method with some modifications [13]. In brief, five microliters of each LAB culture (10^7^ CFU/mL) were spotted on MRS agar in triplicate, and then anaerobically incubated at 37 °C for 48 h. The plates (diameter: 94 mm) were then overlaid with 8 mL of malt extract soft agar (0.5% agar), inoculated with each mold spore suspension (the final concentration was approximately 10^4^ spores/plate), and incubated at 25 °C for 3 days until an even layer of mold was visible. Multispectral images of the plates were captured using a Videometer Lab2 spectral imaging instrument after incubation. The MATLAB 2018b software (MathWorks, Inc., Natick, MA, USA) was utilized to quantify mold growth based on colony size (total number of pixels) [32]. The area (number of pixels) values were then calculated according to the formula:

Area of inhibition zone=Area of total inhibition zone−Area of bacterial spot zone

### 4.3. Determination of Minimal Inoculum Levels of LAB Cultures to Achieve Fungal Inhibition

The minimal inoculum levels needed for the 10 LAB cultures to achieve fungal inhibition was sought by a high-throughput overlay method as previously described with some modifications [12,33]. Different inoculum levels of the ten LAB cultures in MRS broth medium were tested in 24-well microplates (Sigma-Aldrich) with 500 μL of 1.5% MRS agar. Each culture suspension (1 μL) with different concentrations (10^3^, 10^4^, 10^5^, 10^6^, and 10^7^ CFU/mL) was spotted on the surface center of each well. After anaerobic incubation for 48 h at 37 °C, the wells were covered with a 100 μL layer of 0.5% malt extract soft agar, which was then inoculated with the specific molds to a final concentration of 1.0 × 10^3^ spores/mL (100 spores/well). The 24-well microplates were subsequently incubated at 25 °C for a maximum of 8 days. Control groups were prepared similarly to the wells inoculated with molds but without LAB cultures. Evaluation of results was conducted through visual assessment. The growth inhibition test was performed in triplicate for each mold strain.

### 4.4. Sensitivity to LAB in Yoghurt Serum

The sensitivity of these dairy-associated *Penicillium* strains towards different preparations of *L. rhamnosus* LRH01 and *L. plantarum* LP01 in yoghurt serum was assessed through evaluating the direct interactive effect of molds with the C-fermentates and CF-fermentates using an agar spot method. The sensitivities of the molds towards the volatiles generated by the two LAB cultures were evaluated in a “Plate-on-Plate” test system (Figure 1) without direct contact between the molds and the bacterial cultures [14].

A transparent yoghurt surrogate, yoghurt serum, was used as in this assay and prepared using centrifugation (4400× *g*, 30 min, 4 °C), followed by filtration through a 0.22 μm pore size filter for sterilization (Syringe Filter Q-Max, Frisenette ApS, Knebel, Denmark). To obtain LAB C-fermentates, the yoghurt serum was inoculated with *L. rhamnosus* LRH01 and *L. plantarum* LP01 (10^7^ CFU/mL) in 250 mL Duran BlueCap bottles, respectively, and then fermented at 37 °C for 48 h. CF-fermentates were prepared using centrifugation (4400× *g* for 20 min at 4 °C), followed by filtration (0.45 μm pore size filter, Syringe Filter Q-Max, Frisenette ApS, Knebel, Denmark). Un-inoculated yoghurt serum stored at 37 °C for 48 h served as a control group.

#### 4.4.1. Interaction with C-Fermentates

To prepare the agar plates (1% agar), the C-fermentates of *L. rhamnosus* LRH01 and *L. plantarum* LP01, as well as the control group (prepared as described above), were warmed in a 48 °C water bath. These samples were then mixed with melted, tempered agar and subsequently poured onto the plates. Sensitivity towards the C-fermentates was assessed by spotting 10 μL of each spore suspension (1.0 × 10^4^ spores/mL) in triplicate on the C-fermentates agar plates. The agar plates prepared by mixing un-inoculated MRS broth with agar and inoculated with each mold served as the control groups. Multispectral images of the plates after incubation for 5 days at 25 °C were captured as described in Section 4.2. The MATLAB 2018b software (MathWorks, Inc., Natick, MA, USA) was utilized to quantify mold growth based on colony size (total number of pixels). The Inhibitory Effect (%) was calculated using the following formula:

$$Inhibitory\;Effect\left(\%\right)=\frac{P_{control}-P_{sample}}{P_{control}}\times 100$$

where P indicates the total number of pixels of each colony in either control or sample groups.

#### 4.4.2. Interaction with CF-Fermentates

The CF-fermentate agar plates (1% agar) were prepared following the method described for the preparation of the C-fermentate agar plates. The sensitivity of molds towards the CF-fermentates was calculated following the procedure outlined in Section 4.4.1.

#### 4.4.3. Contribution of Volatiles to the Antifungal Activity

A yoghurt agar plate was inoculated with 10 μL of each mold spore suspension at 1.0 × 10^4^ spores/mL (100 spores/spot) in triplicate. The LAB C-fermentate agar plate (prepared as described above) was positioned upside down on the yoghurt agar plate, and the two plates were securely sealed together using parafilm. The inhibitory effect was assessed after incubation for 5 days at 25 °C following the procedure outlined in Section 4.4.1.

### 4.5. Manganese Depletion Test

Commercial plain yoghurt was inoculated with *L. plantarum* LP01 and *L. rhamnosus* LRH01, reaching a final concentration of 10^7^ CFU/mL for each culture. The inoculated yoghurt was then divided into four flasks, to which different concentrations of manganese (0.001 mM, 0.01 mM, and 0.1 mM) were added. A control group containing yoghurt without any LAB cultures or manganese was also included. Yoghurt agar plates (1% agar) were prepared by mixing the yoghurt samples with melted, tempered agar. Subsequently, 10 μL of a spore suspension containing each of the five *Penicillium* strains at a concentration of 1.0 × 10^4^ spores/mL (100 spores/spot) were spotted in triplicate on these yoghurt agar plates. After a 5-day incubation period at 25 °C, multispectral images of the yoghurt agar plates with the spotted molds were captured using a Videometer Lab2. The quantification of mold growth was performed following the procedure described in Section 4.4.1.

### 4.6. A Challenge Test-Yoghurt Production and Biopreservation

#### 4.6.1. Yoghurt Production

Yoghurt production was conducted by Arla Foods (Viby, Denmark) as follows: milk was heated at 85 °C for 30 min first, then rapidly cooled down to 43 °C. In the negative control group, only the starter culture was added into the milk; in the case of experimental groups, both starter culture and bioprotective cultures (10^7^ CFU/mL, *L. rhamnosus* LRH01 and *L. plantarum* LP01) were added into the milk; in the positive control groups, instead of the tested bioprotective cultures, LR4PD (a commercial culture Lyofast composed of *L. rhamnosus* strains) was used as the positive culture and added into the milk. Then the milk samples were fermented at 43 °C until the pH reached 4.5 ± 0.05. After homogenization, the yoghurt samples were cooled down for 20 min to around 22 °C, and then stored at 4 °C. The following day, the yoghurt samples were taken for analysis.

#### 4.6.2. Yoghurt Contamination and Biopreservation Test

The test was carried out using 6-well microplates [34,35]. The yoghurt sample prepared with *L. plantarum* LP01 or *L. rhamnosus* LRH01, respectively, was distributed into each well of the 6-well microplates in triplicate. Then 10 μL of spore suspension (1.0 × 10^3^ spores/mL) of each mold was spotted on the center of each well. Results were determined by visually evaluating mold growth after incubation for up to 40 days at 7 °C compared with the negative control groups. The results were expressed as the number of days to visible mold growth.

### 4.7. Statistical Analyses

The results obtained in this study were presented as the mean ± standard deviation (SD) of three replicates. Statistical analyses were conducted using GraphPad Prism 9.0.2 (GraphPad Software, Inc., San Diego, CA, USA). The data were subjected to one-way analysis of variance (ANOVA) analysis followed by Tukey’s multiple-comparison post hoc test to determine any significant differences. A significance level of *p* < 0.05 was used to declare statistical significance.

## 5. Conclusions

*L. rhamnosus* LRH01 and *L. plantarum* 01 exhibited potent antifungal activity at low levels against five common dairy-associated *Penicillium* strains in laboratory media, and were selected among other strains for further study. In yoghurt serum, the *Penicillium* strains were very sensitive to the cell-containing fermentates of the two cultures, but the removal of cells from fermentates resulted in a significant decrease in antifungal activity. The volatiles exerted even more moderate antifungal effects in this system. Manganese depletion was demonstrated to be a key inhibitory mechanism of the tested LAB cultures in a plain yoghurt matrix since the addition of manganese partly or fully restored the growth of the *Penicillium* strains. In simulated product challenge tests, where the strains were added together with the starter cultures, *L. rhamnosus* LRH01 displayed a strong inhibitory effect in yoghurt, indicating the potential for yoghurt biopreservation. The antifungal activity of *L. plantarum* 01 in yoghurt, on the other hand, was much weaker than observed previously in the laboratory media, emphasizing that the interaction between LAB and spoilage fungi is impacted by a wide range of factors and care should therefore be taken to understand the inhibitory mechanisms in different settings.

## Figures and Tables

**Table 1:** Ten LAB strains and five dairy spoilage-associated *Penicillium* strains used in this study.

Organism	Abbreviation	Source	Provider
Bacteria			
*Lacticaseibacillus rhamnosus* LRH01	*L. rhamnosus* LRH01	Dairy	SACCO
*Lacticaseibacillus rhamnosus* LRH05	*L. rhamnosus* LRH05	Dairy	SACCO
*Lacticaseibacillus rhamnosus* LRH14	*L. rhamnosus* LRH14	Dairy	SACCO
*Lacticaseibacillus rhamnosus* LRH16	*L. rhamnosus* LRH16	Cereals	SACCO
*Lacticaseibacillus rhamnosus* LRH43	*L. rhamnosus* LRH43	Dairy	SACCO
*Lactiplantibacillus plantarum* LP01	*L. plantarum* LP01	Dairy	SACCO
*Lactiplantibacillus plantarum* LP37	*L. plantarum* LP37	Cereals	SACCO
*Lactiplantibacillus plantarum* LP48	*L. plantarum* LP48	Meat	SACCO
*Lacticaseibacillus paracasei* LPC44	*L. paracasei* LPC44	Dairy	SACCO
*Lacticaseibacillus paracasei* LPC46	*L. paracasei* LPC46	Dairy	SACCO
Mold			
*Penicillium crustosum* 01180001	*P. crustosum* 01180001	Yoghurt/Skyr	Arla
*Penicillium commune* ISI2	*P. commune* ISI2	Greek yoghurt	ISI
*Penicillium glabrum* ISI3	*P. glabrum* ISI3	Crème fraiche 18%	ISI
*Penicillium solitum* ISI5	*P. solitum* ISI5	Crème fraiche 30%	ISI
*Penicillium palitans* PPa01	*P. palitans* PPa01	Rahka (Finnish quark)	SACCO

Note: All the LAB strains were obtained from SACCO S.r.l company (Cadorago, Italy) and incubated in an MRS broth/agar or yoghurt serum at 37 °C; the spoilage mold strains isolated from freshly fermented dairy products were provided by Arla Foods (Viby, Denmark), ISI Food Protection ApS (Aarhus, Denmark) and SACCO S.r.l (Italy), respectively. These molds were incubated in MEA/MEB at 25 °C.

**Table 2:** Minimal inoculum levels (CFU/mL) of ten LAB cultures to achieve growth inhibition of five *Penicillium* strains after incubation in MRS medium for 3 days at 25 °C.

LAB cultures/Molds	*P. crustosum* 01180001	*P. commune* ISI2	*P. glabrum* ISI3	*P. solitum* ISI5	*P. palitans* PPa01
*L. rhamnosus* LRH01	10^5^	10^5^	10^4^	10^4^	10^4^
*L. rhamnosus* LRH05	10^5^	10^6^	10^5^	10^5^	10^5^
*L. rhamnosus* LRH14	10^6^	10^7^	10^6^	10^6^	10^5^
*L. rhamnosus* LRH16	10^6^	10^7^	10^5^	10^5^	10^5^
*L. rhamnosus* LRH43	10^6^	10^7^	10^6^	10^6^	10^5^
*L. plantarum* LP01	10^4^	10^6^	10^5^	10^4^	10^4^
*L. plantarum* LP37	10^5^	10^7^	10^6^	10^5^	10^5^
*L. plantarum* LP48	10^7^	10^7^	10^7^	10^7^	10^7^
*L. paracasei* LPC44	10^7^	10^6^	10^6^	10^6^	10^7^
*L. paracasei* LPC46	10^7^	10^7^	10^7^	10^7^	10^7^

Note: The minimal inoculum levels of the LAB cultures were shown in the table: 10^4^ CFU/mL; 10^5^ CFU/mL; 10^6^ CFU/mL; 10^7^ CFU/mL. The results were obtained after incubation for 3 days at 25 °C in laboratory media using the high-throughput overlay method in 24-well microplates.

## Data Availability

The data presented in this study are available on request from the corresponding authors.
